# A Non-Climacteric Fruit Gene *CaMADS-RIN* Regulates Fruit Ripening and Ethylene Biosynthesis in Climacteric Fruit

**DOI:** 10.1371/journal.pone.0095559

**Published:** 2014-04-21

**Authors:** Tingting Dong, Guoping Chen, Shibing Tian, Qiaoli Xie, Wencheng Yin, Yanjie Zhang, Zongli Hu

**Affiliations:** 1 Bioengineering College, Chongqing University, Chongqing, People’s Republic of China; 2 The Institute of Vegetable Research, Chongqing Academy of Agricultural Sciences, Chongqing, People’s Republic of China; Instituto de Biología Molecular y Celular de Plantas, Spain

## Abstract

MADS-box genes have been reported to play a major role in the molecular circuit of developmental regulation. Especially, *SEPALLATA* (*SEP*) group genes play a central role in the developmental regulation of ripening in both climacteric and non-climacteric fruits. However, the mechanisms underlying the regulation of *SEP* genes to non-climacteric fruits ripening are still unclear. Here a *SEP* gene of pepper, *CaMADS-RIN*, has been cloned and exhibited elevated expression at the onset of ripening of pepper. To further explore the function of *CaMADS-RIN*, an overexpressed construct was created and transformed into *ripening inhibitor* (*rin*) mutant tomato plants. Broad ripening phenotypes were observed in *CaMADS-RIN* overexpressed *rin* fruits. The accumulation of carotenoid and expression of *PDS* and *ZDS* were enhanced in overexpressed fruits compared with *rin* mutant. The transcripts of cell wall metabolism genes (*PG*, *EXP1* and *TBG4*) and lipoxygenase genes (*TomloxB* and *TomloxC*) accumulated more abundant compared to *rin* mutant. Besides, both ethylene-dependent genes including *ACS2*, *ACO1*, *E4* and *E8* and ethylene-independent genes such as *HDC* and *Nor* were also up-regulated in transgenic fruits at different levels. Moreover, transgenic fruits showed approximately 1–3 times increase in ethylene production compared with *rin* mutant fruits. Yeast two-hybrid screen results indicated that CaMADS-RIN could interact with TAGL1, FUL1 and itself respectively as SlMADS-RIN did in vitro. These results suggest that *CaMADS-RIN* affects fruit ripening of tomato both in ethylene-dependent and ethylene-independent aspects, which will provide a set of significant data to explore the role of *SEP* genes in ripening of non-climacteric fruits.

## Introduction

The ripening of fleshy fruit is a developmental biochemical process including numerous metabolic changes. These changes not only make fruit assisting in seed dispersal, but also provide essential nutrition for human and animal diets [1]–[3]. Classically, two classes of fruits have been recognized. In climacteric fruits, such as tomato (*Solanum lycopersicum*) and banana (*Musa acuminata*), ethylene synthesis and respiration increased dramatically at the onset of ripening. While in non-climacteric fruits, such as strawberry (*Fragaria* × *ananassa*) and pepper (*Capsicum annuum*), these changes are not found [2]. However, these two distinct programs usually result in similar ripening-related changes, including changes in color, flavor, aroma and nutrition [1], [2].

In climacteric fruits, ethylene plays an important role in triggering the onset of ripening and is an essential factor for the ripening process [4], [5]. Both the functional ethylene synthesis and the ability of ethylene perception are necessary for fruit ripening. For ethylene synthesis, the expression of *ACO1* (*1-aminocyclopropane-1-carboxylic acid oxidase 1*) and *ACO3* (*1-aminocyclopropane-1-carboxylic acid oxidase 3*) are both significantly increased at the onset of fruit ripening [6]. It has been revealed that ethylene production and fruit ripening are strongly inhibited in *ACS2* (*1-aminocyclopropane-1-carboxylic acid synthase 2*) RNAi transgenic tomato fruits [6]. For ethylene perception and response, it is generally considered that *E4* and *E8* are two classical genes which are involved in fruit ripening. The promoter of *E8* has been characterized and is widely used to drive the expression of exogenous genes in transgenic tomato fruits [7]–[9].

Tomato is generally considered to be a model plant for studying climacteric fruit ripening. To date, a wide range of studies have been performed to uncover the mechanism of fruit ripening of tomato, and a cascade of transcription regulators acting upstream of the ethylene pathway has been revealed [2], [10]. In recent years, MADS-box genes have been reported to play a major role in the molecular circuit of developmental regulation [2], [11]–[14]. Several MADS-box genes have been identified and demonstrated to be involved in fruit development in tomato fruit [10], [15]. The antisense suppression of *TAGL1* (*TOMATO AGAMOUS-LIKE 1*) results in ripening inhibition and pericarp thickness reduction [13]. *FUL1* and *FUL2* are orthologs of *Arabidopsis FUL* (*FRUITFULL*) gene,both of which are related to fruit ripening [15], [16]. And recently we reported a *SEP* group MADS-box gene, *SlMADS1* which regulated fruit ripening as an inhibitor [17].

In non-climacteric fruits, ripening is thought to be ethylene independent [2]. However, ethylene may also play roles in ripening of non-climacteric fruits. For instance, grapes have been reported to contain a functional network of ethylene signaling at the onset of ripening [18]. A transient increase of endogenous ethylene production occurs before veraison of grape, and during this stage diameter, acidity and anthocyanin change expeditiously [18]. It has also been reported that the expression of *ACO1* in pineapple is induced in ripening fruit tissue [19]. Furthermore, as well as what occurs during climacteric fruit ripening, there is an increasing synthesis of receptors (i.e. *FaETR1* and *FaERS1*) concomitant with the increased synthesis of ethylene in strawberries [20]. Nevertheless, to date, the molecular regulation of ripening in non-climacteric fruits and the relationship between ethylene and non-climacteric fruits is still unclear.

Prior studies indicated that *SEP* genes played a central role in the developmental regulation of ripening in both climacteric and non-climacteric fruits [21]. The regulation of *SEP* genes to the ripening of climacteric fruits is now well established. *SlMADS-RIN* is a typical *SEP* group gene which regulates tomato ripening, including both ethylene-dependent and ethylene-independent ripening pathways [12]. To date, the transcriptional cascade downstream from *SlMADS-RIN* has been well researched. *SlMADS-RIN* has been reported to control fruit softening, carotenoid accumulation, ethylene production and ethylene perception during ripening [22]–[24]. Besides, SlMADS-RIN interacts with other MADS-box proteins such as TAGL1, FUL1 and FUL2 which involved in fruit ripening, in vitro [16], [25]. In addition, four *SEP* group genes which are highly expressed in fruit have been cloned in banana and *MaMADS2* was reported to act in the pulp upstream of the increase in ethylene production similarly to *SlMADS-RIN* [26]. Suppression of the homeologous *SEPALLATA1*/*2*-like genes in the fleshy fruit apple (*Malus* × *domestica*) led to greatly reduced fruit flesh. Furthermore, like *SlMADS-RIN* gene in tomato, *MADS9* gene acts as a transcriptional activator of the ethylene biosynthesis enzyme, 1-aminocyclopropane-1-carboxylate (ACC) synthase 1 [27]. Nevertheless, the mechanisms underlying the regulation of *SEP* genes to non-climacteric fruits ripening are still unclear. Therefore, in this study, a *SEP* gene which is a potential ortholog to *SlMADS-RIN* has been cloned from a non-climacteric fruit pepper (*Capsicum annuum* L. cv. Bukang). And its function in complementing the *rin* tomato mutant has been examined.

## Results

### Molecular Characterization of *CaMADS-RIN*

The full-length cDNA of *CaMADS-RIN* was cloned previously by our laboratory from pepper and deposited into genbank (accession number: DQ999998). Gene sequence analysis showed that *CaMADS-RIN* contained an ORF of 732 bp, a 5′-UTR (untranslated region) of 74 bp and a 3′-UTR (untranslated region) of 236 bp. The predicted CaMADS-RIN protein had 243 amino acids with an estimated molecular mass of 28 kD. Alignment analysis of amino acid sequences of MADS-box genes exhibited that *CaMADS-RIN* had conserved MADS-box domains (MADS domain, I domain and K domain) and its C-terminal region was highly divergent from other MADS-box sequences. Additionally, phylogenetic analysis revealed that *CaMADS-RIN* belonged to the *SEP* clade and showed the highest similarity to *SlMADS-RIN* (Figure S1).

### *CaMADS-RIN* Exhibited Elevated Expression at the Onset of Ripening of Pepper

**Figure 1:**
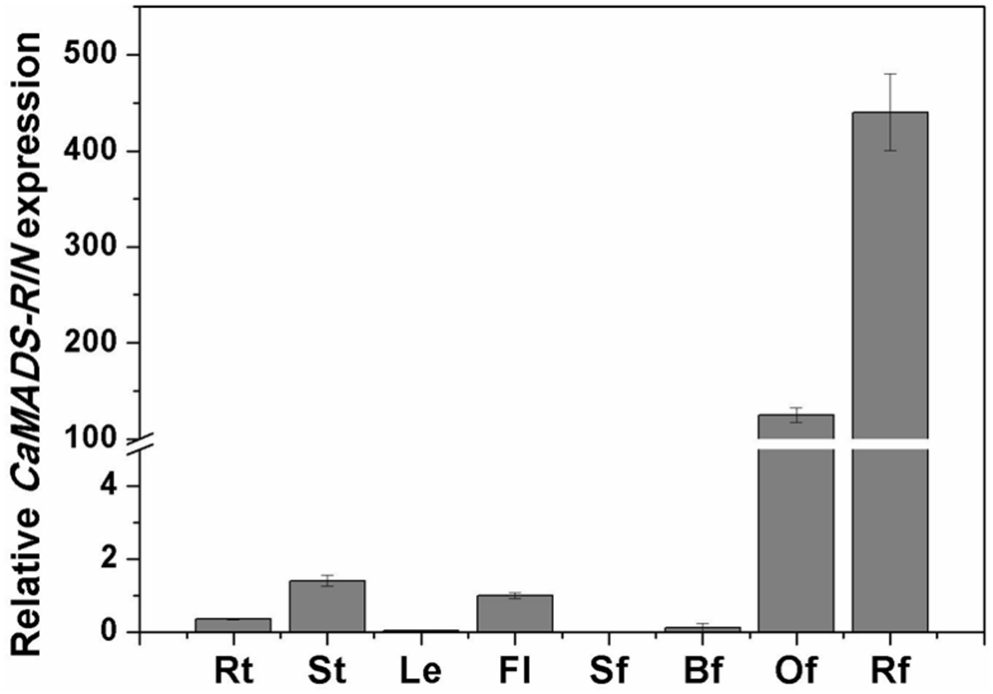
Expression profile of *CaMADS-RIN* in tissues of pepper. The expression of *CaMADS-RIN* in Rt, roots; St, stems; Le, leaves; Fl, flowers; Sf, fruits of 1 cm; Bf, fruits of 6 cm; Of, orange fruits; Rf, red fruits. Expression was determined by Q-RT-PCR as relative quantification. Results are of a representative experiment, and are an average of three repetitions ±SD.

Real-time PCR was performed for analysis the accumulation of *CaMADS-RIN* transcripts in roots, stems, leaves, flowers, and a series of stages of fruits to explore the expression profile of *CaMADS-RIN* in pepper. Low level expression of *CaMADS-RIN* was observed in roots, stems, leaves and flowers (Figure 1). For pepper fruits, the *CaMADS-RIN* expression was low or barely detectable in green fruits, while the transcript showed high expression at the onset of ripening (Figure 1). This expression pattern was similar to the previous reported *SlMADS-RIN* [12], and indicated that *CaMADS-RIN* might be involved in fruit ripening of pepper.

### *CaMADS-RIN* Overexpressed *rin* Fruits Showed Ripening Phenotype

**Figure 2:**
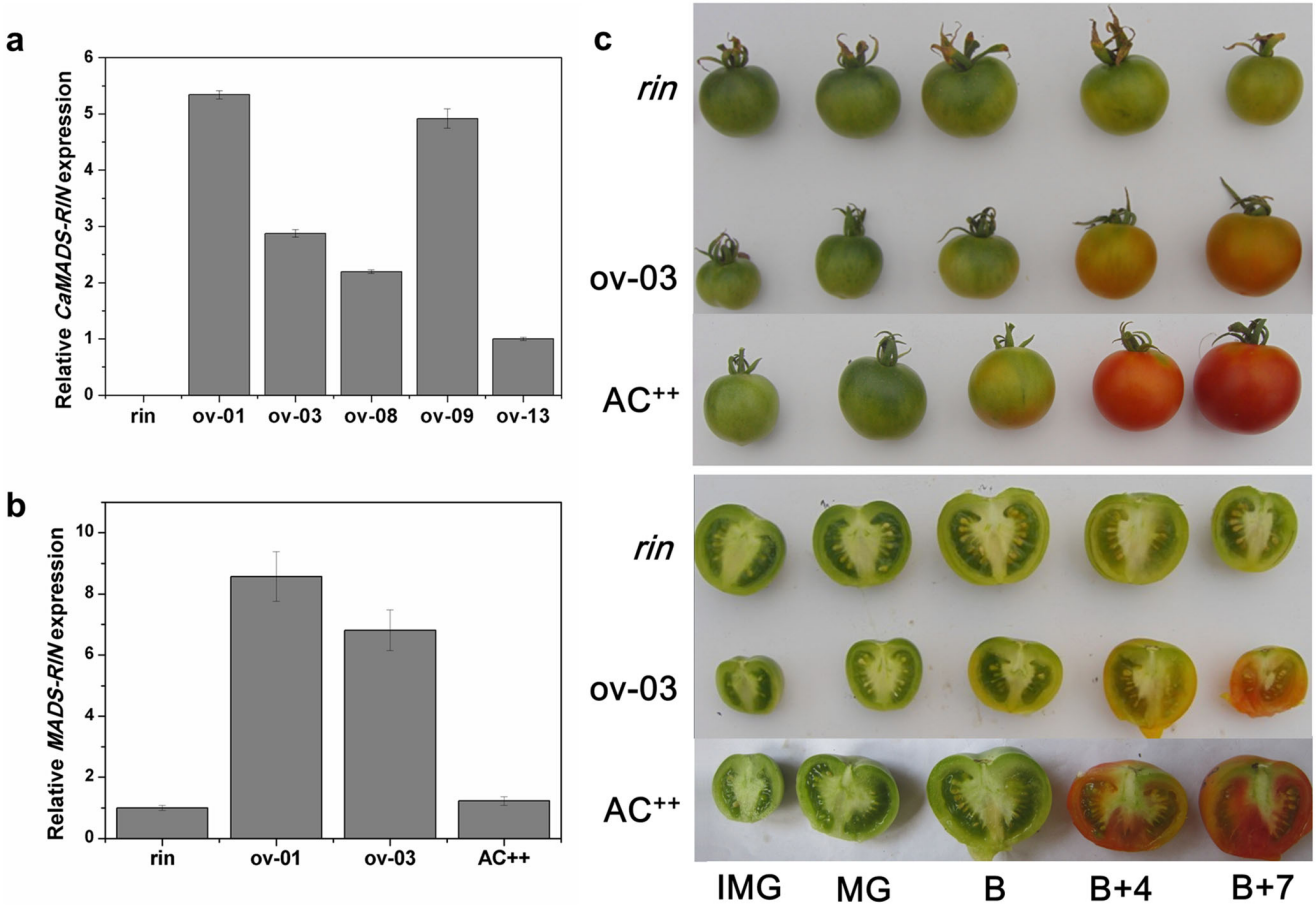
Heterologous expression of *CaMADS-RIN* in *rin* tomato fruit complemented ripening. (a). Expression of *CaMADS-RIN* in overexpressed lines, *rin* mutant and wild type. RNAs were extracted for qPCR assay from B fruits of overexpressed lines and *rin* mutant. Three replications for each sample were performed. (b). Expression of pepper and tomato *MADS-RIN* in overexpressed lines, *rin* mutant and wild type. RNAs were extracted for qPCR assay from B fruits of overexpressed lines, *rin* mutant and wild type. Three replications for each sample were performed. (c). Phenotype of transgenic fruits. Wild type AC^++^, *rin* mutant and transgenic fruits were tagged at anthesis and designated as breaker stage at the same age as wild type fruit which showed the first signs of color change. IMG, MG, B, B+4 and B+7 of *rin* tomato fruits, wild type fruits and *CaMADS-RIN* overexpressed *rin* fruits were shown.

To gain further insight into the function of *CaMADS-RIN*, an overexpressed construct was created and transformed into *rin* mutant tomato plants via *Agrobacterium tumefaciens*–mediated T-DNA transfer. Five independent transgenic lines were produced and integration transgene was confirmed by PCR. Quantitative real-time PCR resulted that abundant *CaMADS-RIN* transcripts were observed in the transgenic lines, while no expression of *CaMADS-RIN* was detected in *rin* mutant (Figure 2a). The expression level of pepper and tomato *MADS-RIN* in wild type, *rin* mutant tomato and transgenic lines was also detected by a pair of conserved primers, CaSlRIN (RT)-F and CaSlRIN (RT)-R, which specially targeted to *CaMADS-RIN*, *SlMADS-RIN* and *SlMADS-RIN* mutant (Figure 2b). The results suggested that transgenic fruits showed approximately 8-fold increases in *MADS-RIN* expression compared to *rin* mutant and wild type.

As shown in Figure S2, no obvious changes of the roots, stems, leaves and flowers were detected in the transgenic plants compared with *rin*. The most striking phenotype of *CaMADS-RIN* overexpressed *rin* lines was that the fruit ripening could be partially restored in transgenic fruits (Figure 2c). Fruits had no color change at the onset of ripening in the *rin* mutant, while the *CaMADS-RIN* overexpressed *rin* fruits had an obvious color change and became yellow-orange at that time (Figure 2c). Flowers were tagged at anthesis, and the time to ripening from anthesis stage was measured for wild type, *rin* and the transgenic *rin* lines. It was observed that ripening time of transgenic *rin* tomato fruits was consistent with wild type, while accelerated about 10 d compared with *rin* mutant (Table 1).

**Table 1:** Days from anthesis to breaker stage for wild type, *rin* and overexpressed lines.

Tomato Lines	Days
Wild Type	38.0±0.50
ov-01	37.8±0.47
ov-03	38.6±0.63
*rin*	48.4±0.51

### Carotenoid Accumulation in Transgenic Fruits were Partially Restored

**Figure 3:**
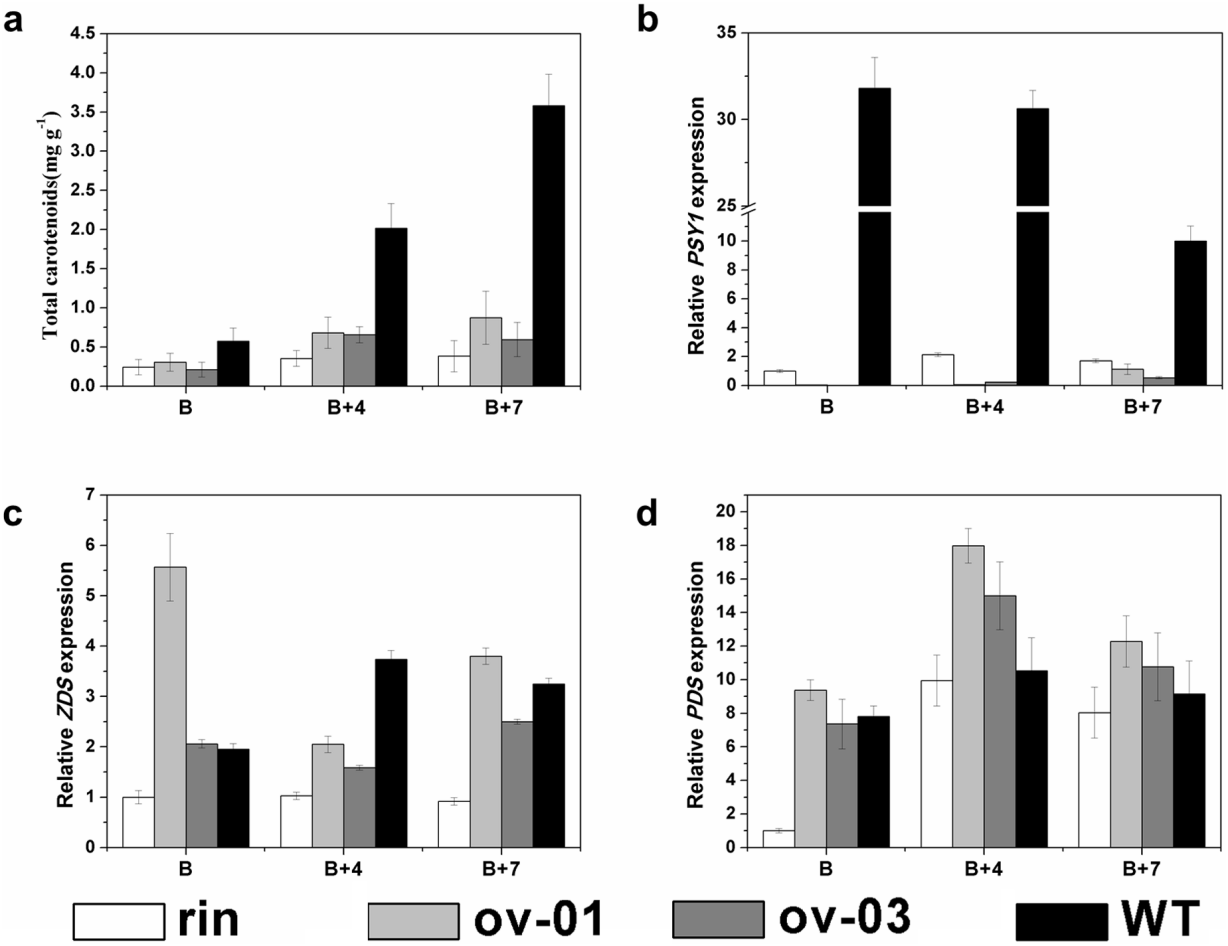
Carotenoid accumulation and carotenoid biosynthesis genes expression in *CaMADS-RIN* overexpressed *rin* and control fruits. (a). Analysis of carotenoid accumulation at B, B+4 and B+7 fruits of transgenic lines (ov-01 and ov-03), *rin* and wild type. Standard error is indicated for a minimum of three fruits per sample. (b). Expression of *PSY1* in B, B+4 and B+7 fruits of transgenic lines (ov-01 and ov-03), *rin* and wild type. (c). Expression of *ZDS* in B, B+4 and B+7 fruits of transgenic lines (ov-01 and ov-03), *rin* and wild type. (d). Expression of *PDS* in B, B+4 and B+7 fruits of transgenic lines (ov-01 and ov-03), *rin* and wild type.

As shown in the Figure 2c, the *CaMADS-RIN* overexpressed *rin* lines developed yellow-orange ripening fruits. Carotenoids are the mainly accumulated color during tomato fruit ripening, thus the total carotenoids in transgenic and control fruits at stages of B (breaker), B+4 (four days after breaker) and B+7 (seven days after breaker) were extracted and determined. The results showed that the accumulation of carotenoid in transgenic lines was higher than *rin* mutant, while was just 30% of wild type tomato AC^++^ (Figure 3a). In order to gain further insight into this phenotype, expression of several carotenoid biosynthesis genes were detected by real-time PCR. The results suggested that *PDS* (*phytoene desaturase*) and *ZDS* (*zeta-carotene desaturase*) were both up-regulated in the overexpressed lines compared with *rin* mutant; the transcripts of *PDS* in B+4 and B+7 stage fruits and *ZDS* in B stage fruits accumulated even higher than wild type (Figure 3c and d). However, for *PSY1* (*Phytone synthease 1*), the rate-limiting enzyme for carotenoid biosynthesis in tomato, the expression was depressed in overexpressed lines compared with *rin* mutant and wild type (Figure 3b).

### Expression of Cell Wall Metabolism-related Genes in *CaMADS-RIN* Overexpressed *rin* Fruits

**Figure 4:**
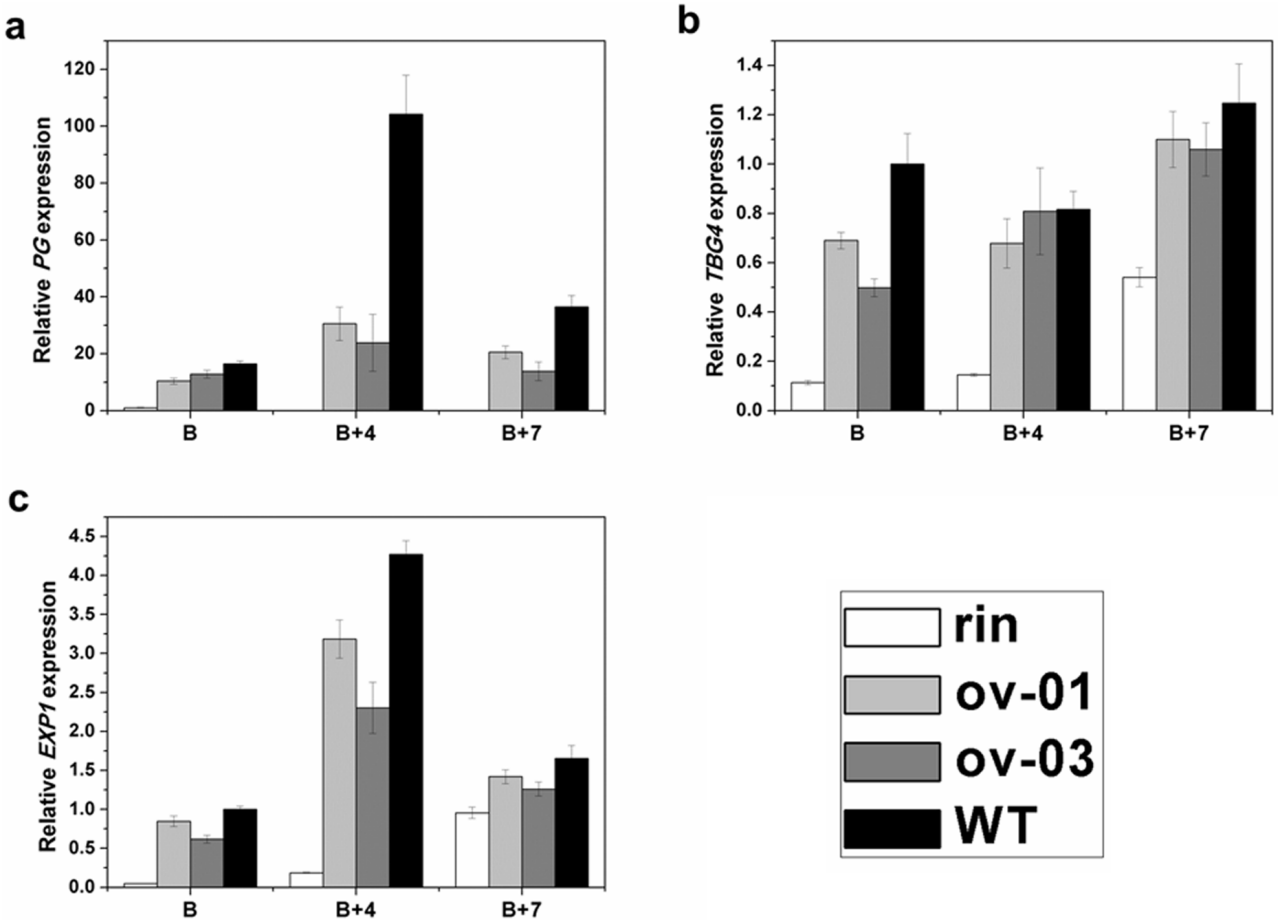
Cell wall metabolism genes in *CaMADS-RIN* overexpressed *rin* and control fruits. (a). Expression of *PG* in B, B+4 and B+7 fruits of transgenic lines (ov-01 and ov-03), *rin* and wild type. (b). Expression of *TBG4* in B, B+4 and B+7 fruits of transgenic lines (ov-01 and ov-03), *rin* and wild type. (c). Expression of *EXP1* in B, B+4 and B+7 fruits of transgenic lines (ov-01 and ov-03), *rin* and wild type.

During the ripening of fruits, the transgenic fruits became as soft as wild type, while the *rin* mutant fruits did not. Thus, the expression of a set of cell wall metabolism-related genes was examined by real-time PCR. The results showed that the critical determinant of cell wall metabolism, *PG* (*polygalacturonase*) [28] was dramatically up-regulated in transgenic fruits compared with *rin*, although it still did not restored to the level of wild type (Figure 4a). Another two genes, *TBG4* (*β-Galactosidase 4*) [29] and *EXP1* (*α-Expansin 1*) [30] showed significant elevation in ripening fruit of transgenic fruits compared to *rin* and nearly recovered to wild type level (Figure 4c, d). It was confirmed that *CaMADS-RIN* overexpression had induced cell wall metabolism in *rin* mutant.

### Expression of Lipoxygenase Genes in *CaMADS-RIN* Overexpressed *rin* Fruits

**Figure 5:**
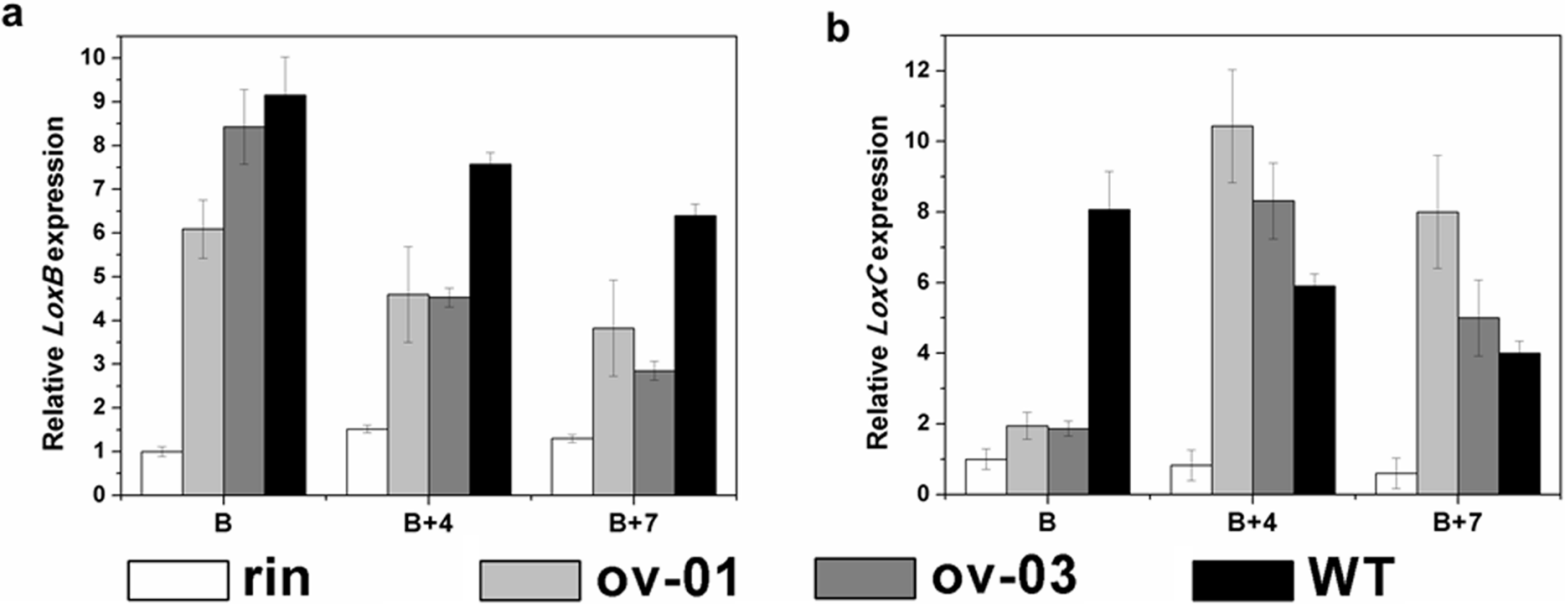
Lipoxygenase genes in *CaMADS-RIN* overexpressed *rin* and control fruits. (a). Expression of *TomLoxB* in B, B+4 and B+7 fruits of transgenic lines (ov-01 and ov-03), *rin* and wild type. (b). Expression of *TomLoxC* in B, B+4 and B+7 fruits of transgenic lines (ov-01 and ov-03), *rin* and wild type.

In order to detect the effect of overexpressing *CaMADS-RIN* on flavor volatiles, two ripening-related genes, *TomloxB* and *TomloxC*, which encode lipoxygenase that transforms polyunsaturated fatty acids into hydroperoxides were detected. The results showed that both of their transcripts were markedly higher in *CaMADS-RIN* overexpressed *rin* fruits than that in *rin* mutant fruits (Figure 5a and b). Furthermore, *TomloxC* even had higher expression level in transgenic fruits at B+4 and B+7 stages compared to wild type (Figure 5b).

### Ethylene Production Ability and Ethylene Biosynthetic Genes were Partially Recovered in Transgenic Lines

**Figure 6:**
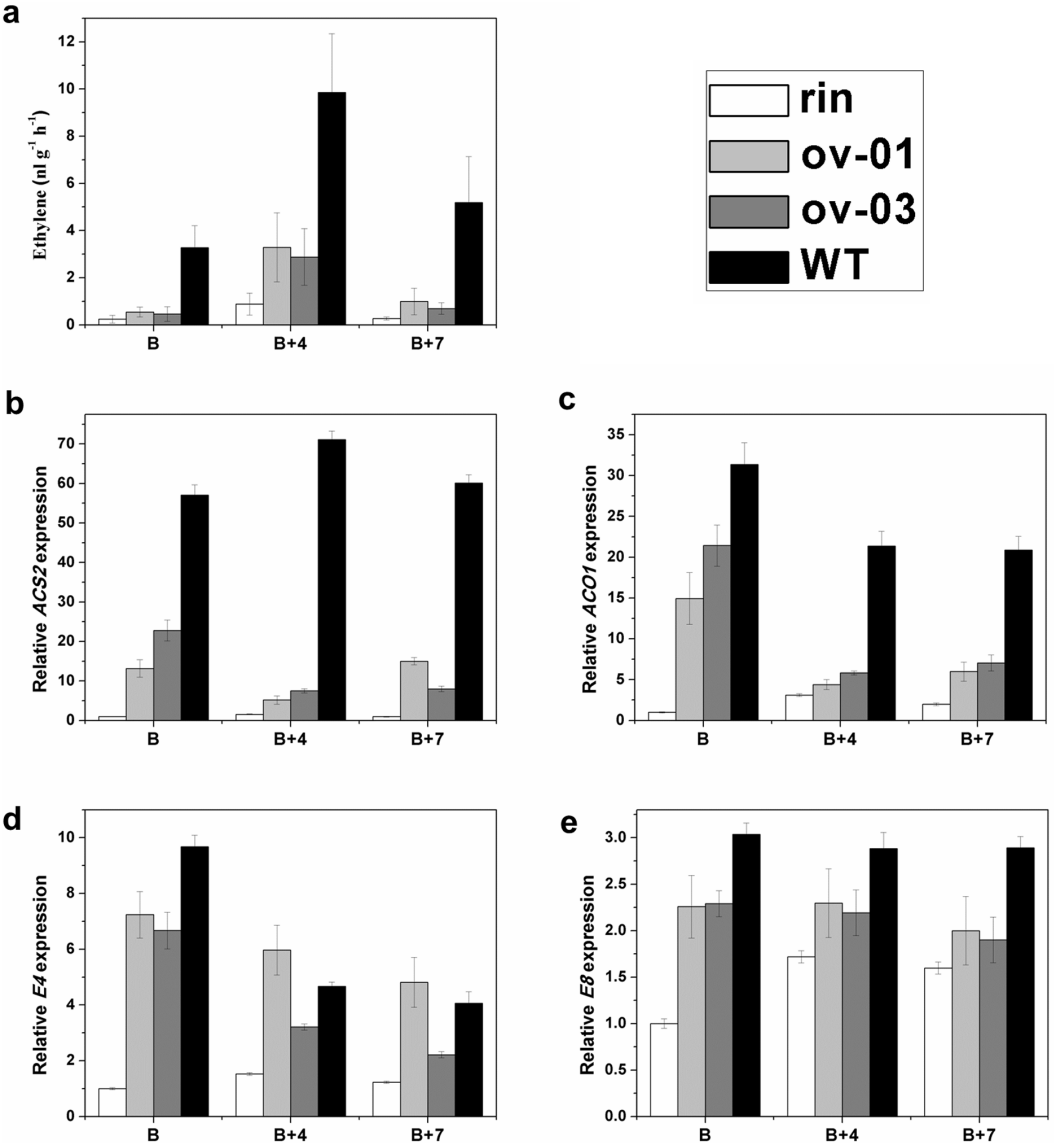
Ethylene production and ethylene biosynthetic and responsive genes expression in control and *CaMADS-RIN* overexpressed lines. (a). Production of ethylene in control and transgenic lines. Fresh fruits of B, B+4 and B+7 were sealed in airtight vials and 1 mL of gas was sampled from the headspace after 24 h. Values represent means of at least three individual fruits. Vertical bars represent standard error. (b) and (c). Expression of ethylene biosynthetic genes, *ACS2* (b) and *ACO1* (c) in control and transgenic lines. RNAs were extracted for qPCR assay from B, B+4 and B+7 fruits of overexpressed lines, *rin* mutant and wild type. Three replications for each sample were performed. (d) and (e). Expression of ethylene responsive genes, *E4* (d) and *E8* (e) in overexpressed lines, *rin* mutant and wild type. RNAs were extracted for qPCR assay from B, B+4 and B+7 fruits of overexpressed lines, *rin* mutant and wild type. Three replications for each sample were performed.

It has been proven that ethylene is an essential factor in triggering the onset of ripening and in regulating the ripening process in climacteric fruits [4], [5]. However, in non-climacteric fruits the role of ethylene is still unclear. Thus, in order to investigate the relation between ethylene and *CaMADS-RIN* of a non-climacteric fruit pepper, we measured ethylene production in *rin* mutant, transgenic and AC^++^ fruits. Although the ethylene production of transgenic lines were still less than AC^++^, transgenic fruits produced approximately 1–3 fold more ethylene than *rin* mutant during fruit ripening (Figure 6a).

To further characterize the molecular mechanism of the ethylene biosynthesis in *CaMADS-RIN* overexpressed *rin* tomatoes, a set of ethylene biosynthetic genes were detected in wild type AC^++^, *rin* mutant and transgenic tomato fruits. Compared with *rin* mutant, *CaMADS-RIN* overexpressed lines exhibited that two ethylene biosynthetic genes, *ACS2* and *ACO1* were dramatically up-regulated in B stage of fruits and slightly up-regulated in B+4 and B+7 stages of fruits (Figure 6b and c), suggesting that, like *SlMADS-RIN*, *CaMADS-RIN* could regulate ethylene production by impacting ethylene biosynthetic genes in tomato.

### Expression of Ripening- and Ethylene-related Genes in *CaMADS-RIN* Overexpressed *rin* Fruits

As shown above, ethylene producing ability was partially recovered in transgenic lines. And it’s reported that a number of genes expression is influenced by ethylene levels. Among them we focused on two genes *E4* and *E8* and the expression of these genes were detected in wild type, *rin* and the transgenic lines. *E4* was markedly increased in transgenic fruits at B and B+4 stages, and slightly induced at B+7 stage (Figure 6d). While the expression of *E8* was significantly higher in all the stages of *CaMADS-RIN* overexpressed *rin* fruits than that in *rin* mutant fruits (Figure 6e). Compared with wild type, *CaMADS-RIN* overexpressed fruits displayed lower expression of both *E4* and *E8* at B stage, while *E4* had higher expression level at B+4 and B+7 stages of ov-01 transgenic fruits (Figure 6).

### Expression of Ethylene-independent Ripening Related Genes in *CaMADS-RIN* Overexpressed *rin* Fruits

**Figure 7:**
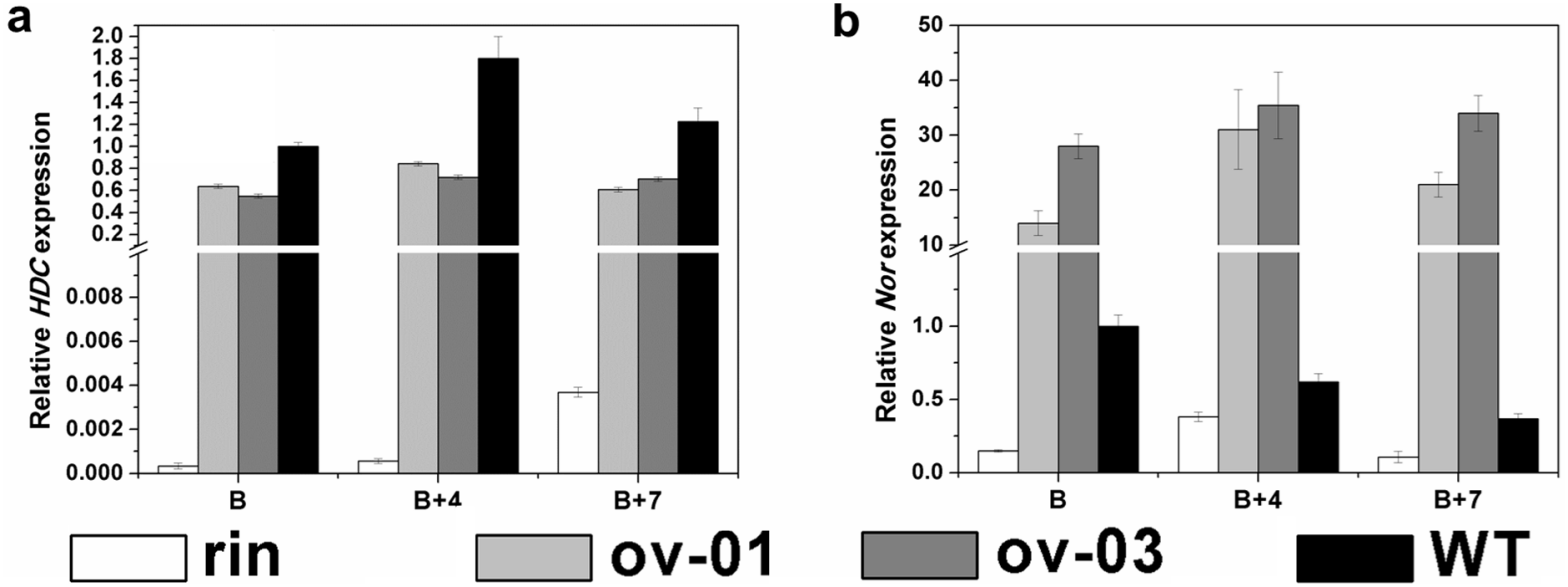
Expression of ethylene-independent genes in overexpressed lines, *rin* mutants and wild type fruits. (a). Expression of a histidine metabolism gene *HDC* in overexpressed lines, *rin* mutants and wild type fruits. (b). Expression of *Nor* in overexpressed lines, *rin* mutants and wild type fruits. RNAs were extracted for qPCR assay from B, B+4 and B+7 fruits of overexpressed lines, *rin* mutant and wild type. Three replications for each sample were performed.

Transcriptome analysis of promoters of differentially regulated genes have provided that *SlMADS-RIN* not only regulates ethylene-dependent aspects but also impacts ethylene-independent aspects of ripening in tomato [31]. In order to detect whether pepper *MADS-RIN* had the same role, we focused on a histidine metabolism gene, *HDC* which is insensitive to ethylene and regulated by *SlMADS-RIN* [32]. The real-time PCR resulted that the transcripts of *HDC* accumulated much higher than *rin*, but was still not rescued to wild type level (Figure 7a). An ethylene-independent ripening related transcript factor Nor, whose promoter is associated with *SlMADS-RIN* [33], [34] was also detected in transgenic fruits. The results showed that *Nor* was markedly up-regulated compared to both *rin* and wild type (Figure 7b).

### CaMADS-RIN Exhibited the Same Interaction Mode as SLMADS-RIN In vitro

**Figure 8:**
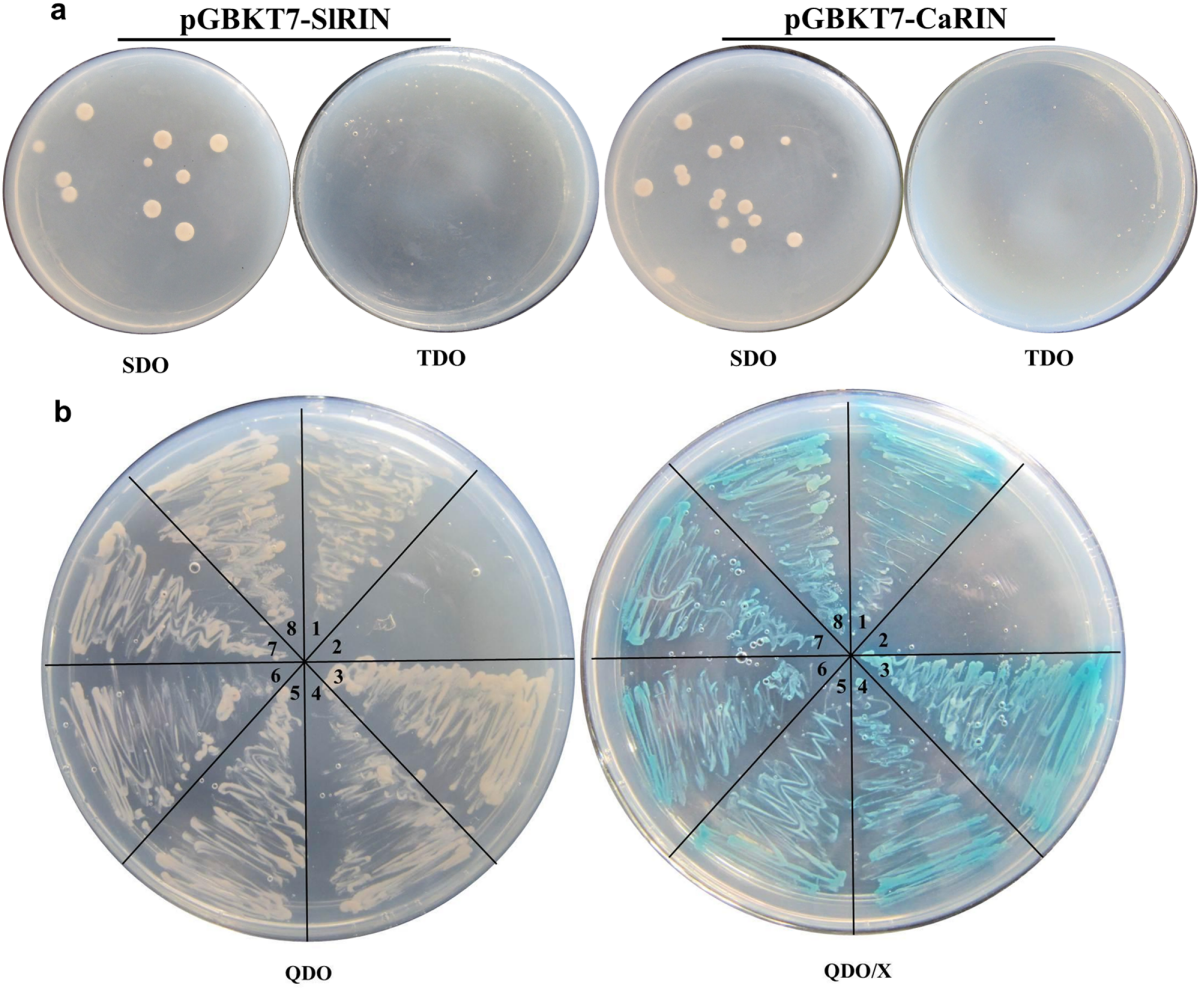
Yeast Two-hybrid Assay for MADS-RINs of Tomato and Pepper and Tomato MADS-box Proteins. (a). Self-activation of pGBKT7-CaRIN and pGBKT7-SlRIN. SDO, SD medium without Trp; TDO, SD medium without Trp, His and Ade. (b). Yeast two-hybrid assay for CaMADS-RIN, SlMADS-RIN and tomato MADS-box proteins. QDO, SD medium without Trp, Leu, His and Ade; QDO/X-α-gal, SD medium without Trp, Leu, His, Ade and with X-α-Gal. 1. pGBKT7–53 & pGADT7-T (positive control); 2. pGBKT7-Lam & pGADT7-T (negative control); 3. pGBKT7-SlRIN & pGADT7-TAGL1 (interaction between SlRIN and TAGL1); 4. pGBKT7-CaRIN & pGADT7-TAGL1 (interaction between CaRIN and TAGL1); 5. pGBKT7-SlRIN & pGADT7-FUL1 (interaction between SlRIN and FUL1); 6. pGBKT7-CaRIN & pGADT7-FUL1 (interaction between CaRIN and FUL1); 7. pGBKT7-SlRIN & pGADT7-SlRIN (interaction between SlRIN and SlRIN); 8. pGBKT7-CaRIN & pGADT7-CaRIN (interaction between CaRIN and CaRIN).

It is reported that hetero- or homo-dimers are often detected in MADS domain proteins [25]. The tomato MADS-box protein SlMADS-RIN whose mutant is *rin* interacts with other MADS-box proteins such as TAGL1, FUL1 and FUL2 which are involved in fruit ripening, in vitro [16], [25]. To further test whether *CaMADS-RIN* could take the place of *SlMADS-RIN* in tomato, yeast two-hybrid assay was performed. The open reading frame of CaMADS-RIN and SlMADS-RIN were amplified and cloned into pGBKT7 as the baits. Self-activation of pGBKT7-CaRIN and pGBKT7-SlRIN were tested and the results are minus (Figure 8a). While the open reading frames of *TAGL1*, *FUL1*, *CaMADS-RIN* and *SlMADS-RIN* were amplified and cloned into pGADT7 as the prey respectively. Figure 8b showed that SlMADS-RIN could interact with TAGL1, FUL1 and itself in vitro. And as expected, CaMADS-RIN exhibited the same interaction mode as SlMADS-RIN had (Figure 8).

## Discussion

### *CaMADS-RIN* Overexpressed *rin* Fruits Exhibited Uncompleted Ripening Phenotype

In this study, we observed that *CaMADS-RIN* overexpressed *rin* fruits appeared yellow-orange (Figure 2c). The accumulation of carotenoid in transgenic lines was higher than that in *rin* mutant, but lower than that in wild type (Figure 3a). It’s reported that the formation of carotenoids is a characteristic of both tomato and pepper fruits, although the other end products that form differ between fruits of the two species [35]. Prior study showed that the expression of *PSY1*, *PDS* and *ZDS* was all reduced dramatically in *rin* mutant and the mutant fruits were green [12], [34]. In our transgenic fruits, *PDS* and *ZDS* expression was both restored or even higher than wild type, which may cause the slightly higher carotenoid accumulation. However, phytone synthease 1 catalyzes a highly influential step for total carotenoid synthesis [36], [37]. A mutation in *PSY1* causes a yellow-fresh phenotype and an absence of carotenoids in ripe fruit [38], [39]. Our result of Figure S3 suggested that *PSY1* had an analogous expression profile in pepper as in tomato, indicating that *PSY1* has the same role in pepper as in tomato. For *PSY1*, a rate-limiting enzyme for carotenoid biosynthesis, its expression was depressed in overexpressed lines (Figure 3b), which might be responsible for the orange color of transgenic fruit.

In addition, compared with wild type, most of the cell wall metabolism and lipoxygenase genes displayed lower expression in transgenic fruits, although they all had higher expression level compared to *rin* (Figure 4 and 5). These results suggested that broad ripening phenotypes of *rin* mutant were not completely rescued by heterologously expressing *CaMADS-RIN*, which might be a consequence of reduced expression of *CaMADS-RIN* gene compared with wild type. Considered this, a pair of conserved primers which specially targeted to *CaMADS-RIN*, *SlMADS-RIN* and *SlMADS-RIN* mutant was designed to detect the expression level of *MADS-RIN* in wild type, *rin* mutant tomato and transgenic lines. The results showed that approximately 8-fold increases of *MADS-RIN* expression in transgenic fruits compared to *rin* mutant and wild type (Figure 2b), indicating that the partial ripening phenotype of transgenic lines were not a consequence of reduced expression of the *CaMADS-RIN* gene but may be a result of other causes.

Recently, two grapevine *SEP* genes, *VviSEP3* and *VviSEP4*, are heterologously expressed in tomato *rin* mutant, which are also just capable of partially complementing the non-ripening phenotype of *rin* [40]. Furthermore, heterologously expression of two banana *SEP* genes, *MaMADS1* and *MaMADS2*, in *rin* tomato fruit could not complement ripening [26]. These results indicate that though *MADS-RINs* in pepper, tomato or other species are highly homologous, there are some different special roles respectively in the regulation of fruit ripening.

### *CaMADS-RIN* is Involved in Both Ethylene-dependent and Ethylene-independent Fruit Ripening

Ethylene plays an essential role during ripening, especially climacteric fruits ripening [5], [6]. The mechanism of ethylene-related ripening in climacteric fruits to date has been well established [2], [6], [12]. Both the normal function of ethylene biosynthesis and the ability of ethylene response are required for ripening process [2], [6], [12]. The fruit of RNAi repression of *ACS2* could not ripen normally [41]. Previous studies also indicated that RNAi inhibition of *ACO1* delays ripening of climacteric fruits [11], [42], [43]. In our study, both of the two ethylene biosynthetic genes were induced significantly in transgenic fruits (Figure 6b and c), indicating that *CaMADS-RIN* regulates ethylene biosynthesis. This result was further confirmed by the higher ethylene production of transgenic fruits. For ethylene response, *E4* and *E8* are two classical genes involved in fruit ripening [44]. Our results showed that the transcripts of the two genes accumulated higher than *rin* mutant (Figure 6d and e), suggesting that besides the functional ethylene synthesis, the ability of ethylene perception and response are also regulated by *CaMADS-RIN*.

Limited information has been published on the mechanism of ethylene-independent fruit ripening. Primary metabolism genes are important members that belong to ethylene-independent group. Among them we focused on *HDC*, whose transcripts are not restored by treatment of fruit with ethylene but involved in fruit ripening [32]. Its transcripts are not detected in tomato leaves and unripe fruit, accumulate during early fruit ripening and then decline [32]. Our real-time PCR showed that *HDC* was significantly up-regulated in transgenic fruits (Figure 7a). Moreover, an ethylene-independent ripening-related transcriptional factor gene, *Nor* belonged to NAC family whose mutant exhibited non-ripening phenotypes [33] was also detected. The results showed that significant induction was detected in transgenic fruits compared with both *rin* and wild type (Figure 7b). In addition, broad ripening phenotypes including carotenoids biosynthesis (Figure 3), cell wall metabolism (Figure 4) and fatty acid-derived flavor compounds metabolism (Figure 5) were all regulated by *CaMADS-RIN*. These results suggest that *CaMADS-RIN* is involved in both ethylene-dependent and ethylene-independent fruit ripening.

### *CaMADS-RIN* May Play an Important Role in Pepper Fruit Ripening

Prior studies have indicated that *rin* mutation displays enlarged sepals and inhibited fruit ripening. This mutant phenotype has been attributed to a function of two MADS-box transcriptional factors, SlMADS-RIN and SlMADS-MC. SlMADS-RIN regulates fruit ripening and SlMADS-MC involves in sepal development [12]. The transcriptional cascade downstream from *SlMADS-RIN* has already been well researched at present. It has been revealed that S*lMADS-RIN* bound to the cis-element of *ACS2* [24], then controlled fruit softening, carotenoid accumulation, ethylene production and ethylene perception [22], [24]. Additional ripening-related genes such as *E4*, *E8*, *PG*, *EXP1* and *TBG4* were also regulated by *SlMADS-RIN* directly [22]. In our study, *CaMADS-RIN* overexpressed *rin* tomato exhibited ripening phenotype (Figure 2). The expression of most target genes of *SlMADS-RIN* was up-regulated in *CaMADS-RIN* overexpressed fruits compared with *rin* mutant (Figures 4, 5, 6, and 7). Additionally, the transgenic lines produced more ethylene than *rin* mutant (Figure 6a). Yeast two-hybrid assay displayed that CaMADS-RIN exhibited the same interaction mode as SlMADS-RIN in vitro (Figure 8). These results suggest that *CaMADS-RIN* plays a positive role in ethylene biosynthesis and fruit ripening of tomato.

Phylogenetic analysis suggested that *CaMADS-RIN* belonged to *SEP* clad (Figure S1). *SEP* genes not only have been reported to have a function on fruit ripening in climacteric fruits, but also play a central role in the developmental regulation of ripening in non-climacteric fruits, such as *FaMADS9* in strawberry [21]. It is reported that *FaMADS9* could lead to the inhibition of normal development and ripening in the petal, achene, and receptacle tissues [21]. Besides, heterologously expressing a grapevine *SEP* gene, *VviSEP4*, was capable to partially complement the non-ripening phenotype of the tomato *rin* mutant [40]. In pepper, MADS-box genes also have been cloned and characterized to be involved in fruit ripening. Two pepper MADS-box genes, *CaMADS1* and *CaMADS6* were reported to play a regulatory role for flower and fruit development through interaction of the two genes products [45]. Our results suggested that the expression pattern of *CaMADS-RIN* in pepper was similar to that of *SlMADS-RIN*, which expressed at the onset of ripening (Figure 1). Combining the prior researches and our results, it could be concluded that *CaMADS-RIN* commits its function in pepper fruit ripening, the same way as in tomato. Although transgenic pepper with reduced expression levels of *CaMADS-RIN* should be required to test this hypothesis, our study about *CaMADS-RIN* will supply a set of significant data for pepper fruit ripening study. Also with the assistant of this study, we can further have more robust conclusions and perform the studies with insight into the role of *SEP* genes in ripening of non-climacteric fruits and the relation between ethylene and non-climacteric fruits ripening.

## Materials and Methods

### Plant Materials and Treatments

In this experiment, *rin* mutant tomato (*Solanum lycopersicon* Mill. cv. *ripening inhibitor*) and wild type tomato AC^++^ (*Solanum lycopersicon* Mill. cv. Ailsa Craig) were used. The plants were planted in greenhouse and watered daily. Transgenic cultures grew under standard greenhouse conditions (16 h-day/8 h-night cycle, 25/18°C day/night temperature, 80% humidity, and 250 µmol m^−2 ^s^−1^ light intensity). Two generations of tomato plants were used in experiments. The plants of first generation (T0) came from tissue culture and plants of the second generation (T1) were from seedlings. Flowers were tagged at anthesis. The ripening stages of tomato fruits were divided according to days after anthesis (dpa) and fruit color. In wild type, IMG (Immature green) fruits were defined as 28 dpa. MG (Mature green) fruits were defined as 35 dpa and were characterized as being green and shiny with no obvious color change. At B (Breaker) stage, fruits color change from green to yellow. After breaker the fruit stages were divided into B+4 (4 days after Breaker) and B+7 (7 days after Breaker). All plant samples were immediately frozen with liquid nitrogen, mixed, and stored at –80°C until further use.

### Phylogenetic Analysis

Full-length cDNA of *CaMADS-RIN* was cloned by screening a cDNA library using the tomato MADS-box gene *SlMADS-RIN* as a probe, and deposited into genbank (accession number: DQ999998). A phylogenetic tree was constructed with the sequence of *CaMADS-RIN* and the other 19 MADS-box genes by MEGA 3.1. The neighbor-joining method contains the following parameters: poisson model,pairwise deletion and bootstrap analysis of 1000 replicates. The numbers at the nodes indicate the bootstrap values. The bar at the bottom indicates the relative divergence of the sequences examined.

### Complementation of *rin* Mutants

Full-length cDNA of *CaMADS-RIN* was amplified with primers CaRINov-F (5′ CGG GAT CCA TGG GTA GAG GGA AAG TAG A 3′) and Oligo d(T)18 (5′ CCC GAG CTC TTT TTT TTT TTT TTT TTT 3′) through high fidelity PCR (Prime STAR™ HS DNA polymerase, Takara, China). Then the amplified products were digested with *Bam*H I and *Sac* I respectively, and linked into pBI121 plasmid at *Bam*H I and *Sac* I restriction sites.

The generated binary plasmids were translated into *Agrobacterium* LBA4404 strain and introduced into *rin* tomato mutant by *Agrobacterium*-mediated transformation described previously [46]. The transgenic plants were detected with primers NPTII-F (5′ GAC AAT CGG CTG CTC TGA 3′) and NPTII-R (5′ AAC TCC AGC ATG AGA TCC 3′). The positive transgenic plants were selected and used for subsequent experiments.

### Quantitative Real-time PCR Analysis

Total RNA from pepper (*Capsicum annuum* L. cv. Bukang), *rin* and transgenic lines were extracted using Trizol (Invitrogen, USA) according to the manufacturer’s instructions. Quantitative real-time PCR analysis was carried out using the CFX96™ Real-Time System (C1000™ Thermal Cycler). All reactions were performed using the SYBR® Premix Ex Taq II kit (TaKARA, China) in a 10 µL total sample volume (5.0µL 2×SYBR Premix Ex Taq, 1.0 µL primers, 1.0 µL cDNA, 3.0µL ddH2O). To remove the effect of genomic DNA and the template from environment, NTC (no template control) and NRT (no reverse transcription control) were performed. Additionally, three replications for each sample were used and standard curves were run simultaneously. Tomato *SlCAC* gene (Table S1) and pepper *β-actin* gene (Table S1) were used as internal standard. The primers CaRIN (RT)-F and CaRIN (RT)-R (Table S1) were used to determine the expression level of *CaMADS-RIN* in pepper, *rin* mutant tomato and transgenic lines. The expression level of *CaMADS-RIN* in wild type, *rin* mutant tomato and transgenic lines were also detected by CaSlRIN(RT)-F and CaSlRIN(RT)-R which is a pair of special primer targeted to *CaMADS-RIN*, *SlMADS-RIN* and *SlMADS-RIN* mutant. Furthermore, the expression levels of fruit ripening and ethylene biosynthesis pathway genes, including *E4* [44], [47], *E8* [48], *PSY1*, *PDS*, *ZDS* [36], [49]–[51], *ACO1* and *ACS2* [6], [52], *PG*, *EXP1* and *TBG4* [28]–[30], *TomloxB* and *TomloxC* [46], [52], [53], *HDC* [32] were determined simultaneously. Primers were shown in table S1.

### Carotenoid Extraction

A 1.0 g sample of each line was cut from pericarp in a 5 mm wide strip around the equator of B, B+4 and B+7 fruits, respectively. Then 10 mL of 60∶40 (v/v) hexane-acetone was added respectively and total carotenoids of wild type (AC^++^), *rin* mutant and transgenic lines fruits were extracted. The extract was centrifuged at 4000 g for 5 min and the absorbance of supernatant was measured at 450 nm. Carotenoid content was calculated with the following equations: total carotenoid mg mL^−1^ = 4*(OD 450)*10 mL/1 g [49], [54]. Three independent experiments were performed for each sample.

### Ethylene Measurements

Fruits of *rin* mutant, AC^++^ and transgenic fruits at B, B+4, B+7 stages were harvested and placed in open 100 mL jars for 3 h to minimize the effect of wound ethylene caused by picking. Jars were then sealed and incubated at room temperate for 24 h, 1 mL of headspace gas was injected into a Hewlett-Packard 5890 series gas chromatograph equipped with a flame ionization detector (FID). Samples were compared with reagent grade ethylene standards of known concentration and normalized for fruit weight [35].

### Yeast Two-hybrid Assay

Yeast two-hybrid was performed using the MATCHMAKER TM GAL4 Two-Hybrid System III according to the manufacturer’s protocol (Clontech). The open reading frame of *SlMADS-RIN* and *CaMADS-RIN* were amplified by PCR respectively with the primer pairs SlMADS-RIN(Y)-F (5′ CCG GAA TTC ATG GGT AGA GGG AAA GTA GA 3′) and SlMADS-RIN(Y)-R (5′ CGC GGA TCC GTC AAA GCA TCC ATC CAG GT 3′); SlMADS-RIN(Y)-F (5′ CCG GAA TTC ATG GGT AGA GGG AAA GTAG 3′) and SlMADS-RIN(Y)-R (5′ CGC GGA TCC GTC AAA GCA TCC ATC CAG G 3′). The PCR products were digested using *Eco*R I and *Sal* I and cloned into the *Eco*R I/*Sal* I site of the pGBKT7 bait vector to obtain the vector pGBKT7-SlRIN and pGBKT7-CaRIN. Then the vectors were translated into Y2HGold. The Y2HGold with baits were plated on SD medium lacking Trp (SDO) and SD medium lacking Trp, His, Ade (TDO) to test self-activation of pGBKT7-SlRIN and pGBKT7-CaRIN. In parallel, the open reading frame of SlTAGL1 and TFUL1 were also amplified by primers SlTAGL1 (Y)-F (5′ CCG GAA TTC ATG GTT TTT CCT ATT AAT C 3′) and SlTAGL1 (Y)-R (5′ CGC GGA TCC GTC AGA CAA GCT GGA GAG G 3′); SlFUL1 (Y)-F (5′ CCG GAA TTC ATG GGA AGA GGA AGA GTC C 3′) and SlFUL1 (Y)-R (5′ CGC GGA TCC GTC ACA GTA TTA TTA GCT G 3′). These products were cloned into the pGADT7 vector, and translated into Y187. In order to test whether SlRIN and CaRIN could interact with each other, these two genes were also cloned into the pGADT7 vector, and translated into Y187. Subsequently, Y2HGold with baits and Y187 with preys were cultured together respectively in 2×YPDA medium for 24 h at 30°C. After that these cultures were cultured on SD medium lacking Trp, Leu (DDO) to select for diploids containing prey and bait vectors. After 2 to 5 days, fresh diploid cells were plated on SD medium lacking Trp, Leu, and His, Ade, with X-α-Gal (QDO/X) to judge the proteins interaction. Plates were incubated for 3 to 7 days at 30°C. An empty prey and bait vector were used as negative controls with each bait and prey construct, respectively. Meanwhile, positive control was transformed and cultured. The assays were repeated at least three times with fresh transformants.

## Supporting Information

Figure S1 **Multiple sequence alignment and phylogenetic analysis of CaMADS-RIN and other known MADS-box proteins.** (a). Multiple sequence alignment of CaMADS-RIN and other MADS-box proteins. Identical amino acids are shaded in black, and similar amino acids are shaded in gray. The MADS box, K box, I region, and C region are identified. (b). Phylogenetic analysis of the CaMADS-RIN and other known MADS-box proteins. CaMADS-RIN is marked with asterisk. Accession numbers and corresponding references for the proteins listed are as follows: AtSEP1 (AED92207.1), AtSEP2 (AEE73791.1), AtSEP3 (AEE30503.1), AtSEP4 (AEC05738), AtAGL24 (AEE84922), SlMADS1 (AY294329), SlMADS-RIN (NP_001233976), TM5 (AGL9_SOLLC), TM29 (NP_001233911), TAG1 (AAA34197), TAGL1 (NP_001234187), FUL1 (NP_001234173), CaMADS-RIN (ABJ98752), CaMADS1 (AF129875), CaJOINTLESS (AFI49342), PPI (ADR83606), PAP3 (ADI58370), PhFBP9 (AF335236_1), PhFBP29 (AF335245_1), PhFBP22 (AF335240_1).
(PDF)

Figure S2 **The phenotype of roots (Rt), stems (St), leaves (Le) and flowers (Fl) in transgenic and** ***rin*** **lines.** Roots, stems and leaves were collected from plants which were flowering; Flowers were photoed at anthesis.
(PDF)

Figure S3 **Expression of** ***CaPSY1*** **in pepper fruits. RNAs were extracted for qPCR assay from a series of fruits in pepper.** Bf, fruits of 6cm; Of, orange fruits; Rf, red fruits. Three replications for each sample were performed.
(PDF)

Table S1 Details of primers for qPCR amplification.
(PDF)
