# Supplementary material for: A Non-Climacteric Fruit Gene CaMADS-RIN Regulates Fruit Ripening and Ethylene Biosynthesis in Climacteric Fruit
Source: PLoS One. 2014 Apr 21;9(4):e95559. doi: 10.1371/journal.pone.0095559 (PMC3994064; doi:10.1371/journal.pone.0095559)
Supplement: Figure S1 — Multiple sequence alignment and phylogenetic analysis of CaMADS-RIN and other known MADS-box proteins. (a). Multiple sequence alignment of CaMADS-RIN and other MADS-box proteins. Identical amino acids are shaded in black, and similar amino acids are shaded in gray. The MADS box, K box, I region, and C region are identified. (b). Phylogenetic analysis of the CaMADS-RIN and other known MADS-box proteins. CaMADS-RIN is marked with asterisk. Accession numbers and corresponding references for the proteins listed are as follows: AtSEP1 (AED92207.1), AtSEP2 (AEE73791.1), AtSEP3 (AEE30503.1), AtSEP4 (AEC05738), AtAGL24 (AEE84922), SlMADS1 (AY294329), SlMADS-RIN (NP_001233976), TM5 (AGL9_SOLLC), TM29 (NP_001233911), TAG1 (AAA34197), TAGL1 (NP_001234187), FUL1 (NP_001234173), CaMADS-RIN (ABJ98752), CaMADS1 (AF129875), CaJOINTLESS (AFI49342), PPI (ADR83606), PAP3 (ADI58370), PhFBP9 (AF335236_1), PhFBP29 (AF335245_1), PhFBP22 (AF335240_1). (PDF) [file pone.0095559.s001.pdf]

Figure S1

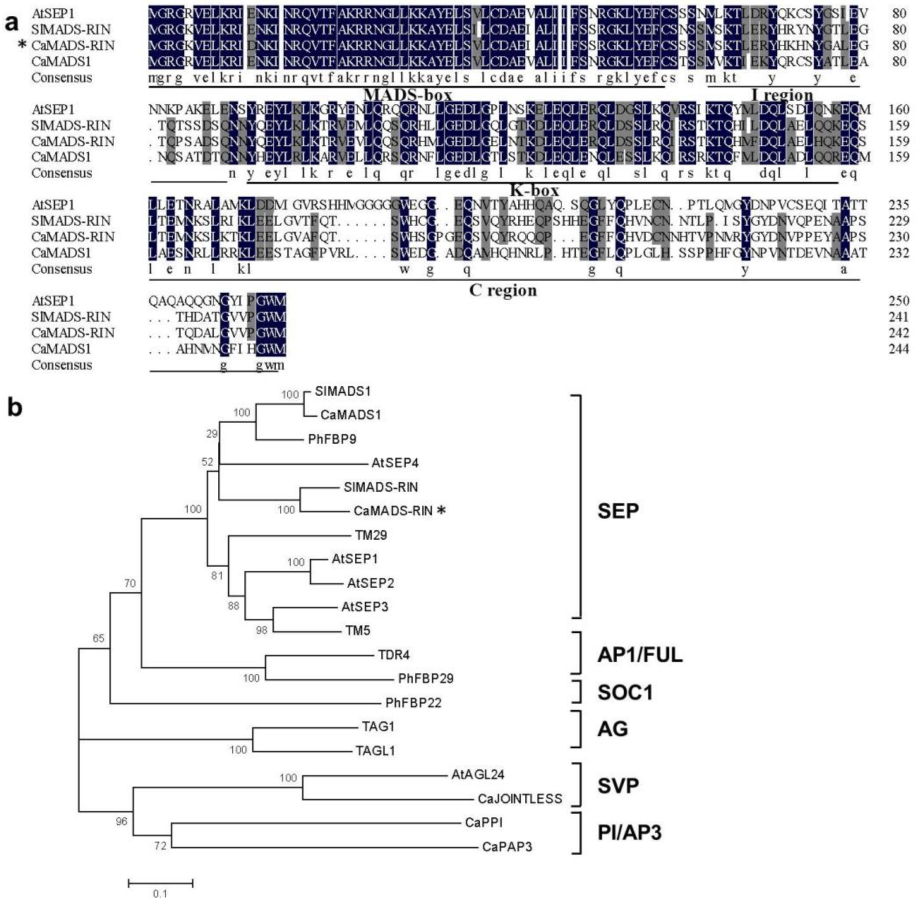

Figure S1 Multiple sequence alignment and phylogenetic analysis of CaMADS-RIN and other known MADS-box proteins. (a). Multiple sequence alignment of CaMADS-RIN and other MADS-box proteins. Identical amino acids are shaded in black, and similar amino acids are shaded in gray. The MADS box, K box, I region, and C region are identified. (b). Phylogenetic analysis of the CaMADS-RIN and other known MADS-box proteins. CaMADS-RIN is marked with asterisk. Accession numbers and corresponding references for the proteins listed are as follows: AtSEP1 (AED92207.1), AtSEP2 (AEE73791.1), AtSEP3 (AEE30503.1), AtSEP4 (AEC05738), AtAGL24 (AEE84922), SlMADS1 (AY294329), SlMADS-RIN (NP_001233976), TM5 (AGL9_SOLLC), TM29 (NP_001233911), TAG1 (AAA34197), TAGL1 (NP_001234187), FUL1 (NP_001234173), CaMADS-RIN (ABJ98752), CaMADS1 (AF129875), CaJOINTLESS (AFI49342), PPI (ADR83606), PAP3 (ADI58370), PhFBP9 (AF335236_1), PhFBP29 (AF335245_1), PhFBP22 (AF335240_1).
