# Supplementary material for: A Non-Climacteric Fruit Gene CaMADS-RIN Regulates Fruit Ripening and Ethylene Biosynthesis in Climacteric Fruit
Source: PLoS One. 2014 Apr 21;9(4):e95559. doi: 10.1371/journal.pone.0095559 (PMC3994064; doi:10.1371/journal.pone.0095559)
Supplement: Figure S2 — The phenotype of roots (Rt), stems (St), leaves (Le) and flowers (Fl) in transgenic and rin lines. Roots, stems and leaves were collected from plants which were flowering; Flowers were photoed at anthesis. (PDF) [file pone.0095559.s002.pdf]

Figure S2

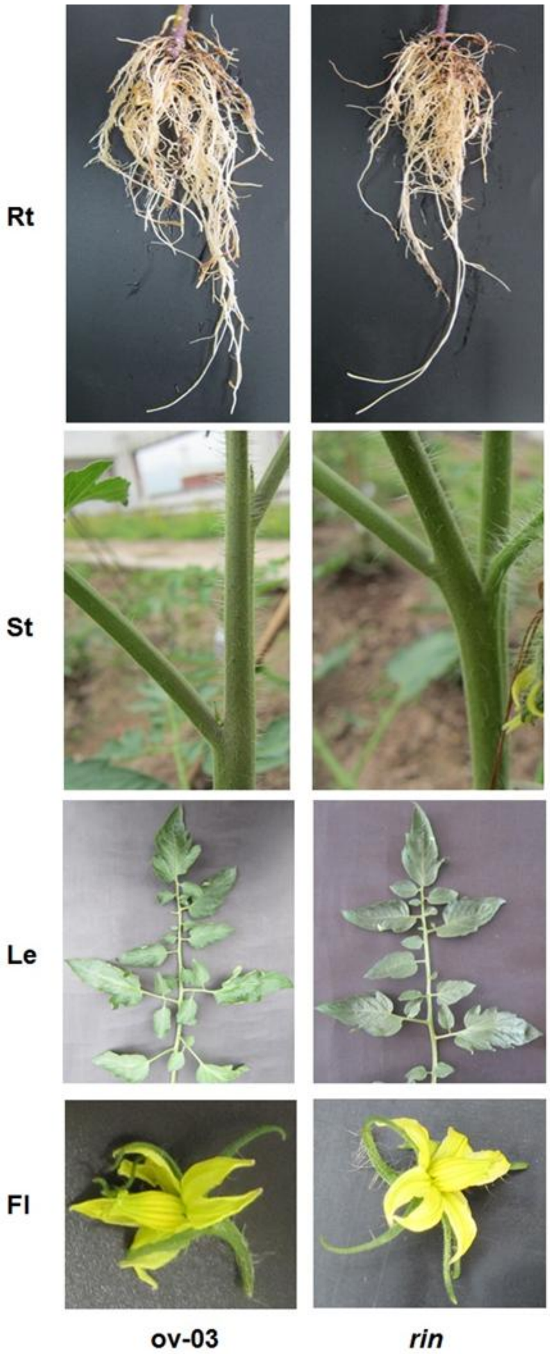

Figure S2 The phenotype of roots (Rt), stems (St), leaves (Le) and flowers (Fl) in transgenic and *rin* lines. Roots, stems and leaves were collected from plants which were flowering; Flowers were photoed at anthesis.
