# Supplementary material for: A Non-Climacteric Fruit Gene CaMADS-RIN Regulates Fruit Ripening and Ethylene Biosynthesis in Climacteric Fruit
Source: PLoS One. 2014 Apr 21;9(4):e95559. doi: 10.1371/journal.pone.0095559 (PMC3994064; doi:10.1371/journal.pone.0095559)
Supplement: Figure S3 — Expression of CaPSY1 in pepper fruits. RNAs were extracted for qPCR assay from a series of fruits in pepper. Bf, fruits of 6cm; Of, orange fruits; Rf, red fruits. Three replications for each sample were performed. (PDF) [file pone.0095559.s003.pdf]

Figure S3

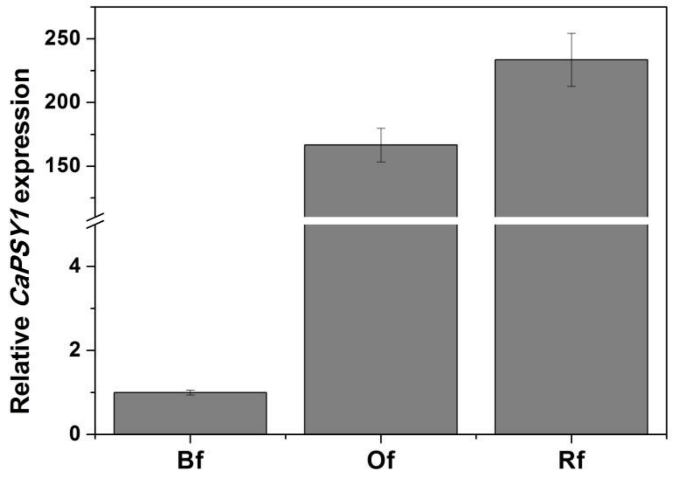

Figure S3 Expression of *CaPSY1* in pepper fruits. RNAs were extracted for qPCR assay from a series of fruits in pepper. Bf, fruits of 6cm; Of, orange fruits; Rf, red fruits. Three replications for each sample were performed.
