# Supplementary material for: A Non-Climacteric Fruit Gene CaMADS-RIN Regulates Fruit Ripening and Ethylene Biosynthesis in Climacteric Fruit
Source: PLoS One. 2014 Apr 21;9(4):e95559. doi: 10.1371/journal.pone.0095559 (PMC3994064; doi:10.1371/journal.pone.0095559)
Supplement: Table S1 — Details of primers for qPCR amplification. (PDF) [file pone.0095559.s004.pdf]

Table S1. Details of primers for qPCR amplification

Primer names	Primer sequences
SlCAC	CCTCCGTTGTGATGTAACTGG
	ATTGGTGGAAAGTAACATCATCG
β-actin	TGCAGGAATCCACGAGACTAC
	TACCACCACTGAGCACAATGTT
CaMADS-RIN	AAACATCATGGCACTCTGGGC
	CGGTGGCACATTATCGTATCC
CaSlMADS-RIN	CAAATAAGGTCAACTAAGACACAAC
	AAAGATTTGTTCATTTCAGTAAGAG
E4	AGGGTAACAACAGCAGTAGCA
	CCCAACCTCCGTCTTCAC
E8	GGCACCATTCAACATACCG
	CTTTCACCGAAGAAGCACG
PSY1	AGAGGTGGTGGAAAGCAA
	TCTCGGGAGTCATTAGCAT
CaPSY1	TAGCACAGGCAGGTCTATCCG
	CCATCTACTAGCTGCGCTCAATT
PDS	GCTTTACCCGCTCCTTTA
	ACCTTGCTTTCTCATCCA
ZDS	GGTGGGTGCTGAAAAAAAT
	GGAAAGCGGAAATCAAGTT
ACO1	ACAAACAGACGGGACACGAA
	CTCTTTGGCTTGAAACTTGA
ACS2	GAAAGAGTTGTTATGGCTGGTG
	GCTGGGTAGTATGGTGAAGGT
LoxB	TGCTACAATGACTTGGGTGAA
	CCTGTCCTGCCTCTACG
LoxC	TTTACTCCGCCCTACACGC
	CCTGAAAGATCGACACCCA
PG	ATACAACAGTTTTCAGCAGTTCAAGT
	GGTTTTCCACTTTCCCCTACTAA
EXP1	CCCTCCTCGCCCTCACTTT
	TCTGATTCCTCCTTGCTTTCG

TBG4	AGTTCTTCTGTTGAATGGGTTCG
	TAGTCTGGCACTTCTTCTCGTTG
HDC	AGAGGAAGAGGTGGTTTCAGGAT
	TGGTTCCAAGGAGTTTAGTTATGC
HB-1	TCTCTGATTTTGACTCCGTTCG
	TTTCATTTCCCCCTGCTCCT
Nor	TAATGATGGGGTCGTCTTTCG
	ATTTTACAGGGCTAACTATTTTTTGC
